# Latent profiles of self-management behavior and associated factors among Chinese patients with ulcerative colitis

**DOI:** 10.3389/fpubh.2026.1749767

**Published:** 2026-04-17

**Authors:** Man Yang, Huiying An, Lei Lei, Yan Chen, Yanyan Wang, Xiaoke Jiang, Xiaoying Luo

**Affiliations:** Henan Provincial People's Hospital, People's Hospital of Zhengzhou University, Zhengzhou, Henan, China

**Keywords:** depression, family communication, latent profile analysis, psychological resilience, self-management behavior, ulcerative colitis

## Abstract

**Background:**

Ulcerative colitis (UC) poses a growing clinical and socioeconomic burden in China. Effective self-management is critical for long-term disease control, yet significant heterogeneity exists in self-management behavior (SMB) among patients. This study aimed to identify latent profiles of SMB and examine associated factors among patients with UC.

**Methods:**

In this cross-sectional study, 415 UC patients completed validated measures including the IBD Self-management Scale (36 items, 7 domains), the Connor–Davidson Resilience Scale−10 (CD-RISC-10), Patient Health Questionnaire-9 (PHQ-9), Family Communication subscale of the Family Assessment Device, and Social Support Rating Scale (SSRS). Latent profile analysis (LPA) was used to identify distinct SMB patterns. Multinomial logistic regression (R3STEP procedure) examined factors associated with profile membership, with the largest class (Low-Monitoring) as the reference.

**Results:**

Three latent profiles were identified: Low-Monitoring (53.97%), characterized by poor symptom surveillance and exercise management; Emotion-Adaptive (37.11%), with strong emotion regulation but limited resource utilization; and High-Engagement (8.92%), demonstrating balanced and proactive self-management across domains. Compared with the Low-Monitoring group, patients in the Emotion-Adaptive and High-Engagement profiles had significantly higher social support (OR = 1.054 and 1.102, respectively; both *p* < 0.01) and lower depressive symptoms (OR = 0.856 and 0.539, respectively; both *p* < 0.01). High-Engagement members also showed higher psychological resilience (OR = 1.112, *p* = 0.009) and better family communication (OR = 1.466, 95% CI: 0.985 2.181, *p* = 0.049), although the latter association was of borderline statistical significance. Rural residence, middle income, and being in clinical remission were associated with lower odds of belonging to more engaged profiles.

**Conclusion:**

Despite the single-center design and a predominantly hospitalized cohort limiting generalizability to outpatient or community populations, this study characterizes the heterogeneity of self-management behavior (SMB) among Chinese patients with UC through latent profile analysis, identifying a behavioral typology that provides a descriptive framework for understanding patient-reported outcomes.

## 1 Introduction

Ulcerative colitis (UC) is an idiopathic inflammatory condition of the colon that results in diffuse friability and superficial erosions on the colonic wall associated with bleeding. It is the most common form of inflammatory bowel disease (IBD) worldwide. UC imposes considerable clinical and socioeconomic burdens. During active disease, patients commonly experience severe abdominal pain, hematochezia, and diarrhea; despite therapeutic advances, a significant proportion of patients face long-term risks of complications such as intestinal obstruction, perforation, or colitis-associated neoplasia (1, 2). Beyond symptoms alone, UC imposes an enormous additional burden on patients' social, professional, and family lives. The UC-LIFE survey (3) revealed that high proportions of patients considered their disease influenced leisure activities (65.1%), recreational or professional activities (57.6%), or relationships with relatives or friends (9.9%). Recurrent hospitalizations and the increasing use of biologic agents have escalated direct medical expenditures; in the Chinese context, the mean per-admission cost for UC has been reported at approximately USD 827 (4). These escalating challenges underscore the urgent need for optimized disease management strategies.

The imperative for patient-centered care in UC is underscored by international initiatives to standardize outcome measurement. Notably, the 2024 Core Outcome Set (COS) for IBD, within which UC is a major subtype, explicitly identifies patient-reported outcomes (PROs) as fundamental treatment targets in both UC clinical trials and routine management (5). Importantly, this consensus expands the conceptual boundaries of PROs beyond traditional domains, such as symptoms, psychological burden, fatigue, and health-related quality of life (HRQoL), to include self-management and autonomy as an independent core PRO domain. Within this framework, self-management behavior (SMB), which refer to the proactive actions patients undertake to manage symptoms, adhere to treatment regimens, and adapt their lifestyles in response to disease demands (6), represent a central element of PROs. They reflect patients' perceived competence in managing their condition in daily life. Conceptually, SMB also constitute the key behavioral pathway linking patients' internal psychological resources to other PRO domains (7). The central role of this behavioral pathway is well supported by behavioral science theories. Social Cognitive Theory positions SMB serve as a dynamic process that bolsters self-efficacy; by successfully executing these behaviors, patients enhance their perceived control and mastery over the disease, which in turn influence health status (8), while Self-Determination Theory emphasizes when SMB are autonomously integrated into a patient's daily lifestyle, they foster sustained adherence and psychological resilience, ultimately securing long-term wellbeing and improved health status (9). Empirical evidence, including studies conducted in IBD populations, is consistent with these theoretical models (10, 11): effective SMB directly contribute to improved disease control in chronic conditions such as UC, while successful disease control experiences, in turn, enhance patients' self-efficacy, forming a self-reinforcing positive feedback loop. Consequently, SMB should be conceptualized as actionable, “process-oriented” levers that exert a direct influence on PROs, including symptom control and health-related quality of life. International evidence also indicates that suboptimal self-management practices are associated with adverse clinical outcomes, underscoring the clinical importance of this behavioral pathway (10).

Despite the critical importance of self-management, empirical evidence reveals that SMB among patients with UC are consistently suboptimal and variably distributed. A qualitative study conducted in East China identified multiple, interconnected obstacles to medication adherence among patients with UC-predominant IBD (12). Such adherence challenges are not region-specific; a cross-cultural comparative study of patients in Japan and the United Kingdom further confirmed that self-reported medication adherence is not only generally suboptimal but also exhibits significant cross-cultural variations in its patterns and influencing factors (13). Dietary practices are frequently guided by beliefs rather than evidence, with up to 80% of patients adopting restrictive elimination diets (14) and may increase the risk of nutritional deficiency (15). Furthermore, symptom monitoring behaviors in UC patients show marked variability across studies, with some cohorts demonstrating limited engagement in structured tracking while others report high levels of proactive self-monitoring (16, 17). This heterogeneity suggests that patients may differ not only in overall engagement but in domain-specific performance (e.g., strong medication adherence coupled with poor emotional coping) (18). Such complexity highlights the limitations of variable-centered analyses and underscores the need for person-centered methods, such as Latent Profile Analysis (LPA). While LPA has been increasingly utilized to uncover self-management heterogeneity in chronic conditions such as diabetes and cancer (19, 20), its application in IBD, and particularly in ulcerative colitis (UC), remains relatively underexplored. UC is characterized by a relapsing-remitting disease course that necessitates complex and ongoing lifestyle adjustments. Patients often adopt unevidenced dietary practices and face substantial challenges with medication adherence, highlighting the need for a nuanced understanding of behavioral patterns in this specific population. By applying LPA to UC patients, the study extends previous methodological approaches into the context of UC, providing the empirical basis for clinicians to replace “one-size-fits-all” advice with targeted, phenotype-driven interventions. The identification of modifiable targets for such interventions is guided by the Middle-Range Explanatory Theory of Self-Management behavior (21). This framework posits that antecedents such as psychological resilience (22), family functioning (23), and social support (24) operate as upstream predictors that shape self-management processes.

In the context of UC, self-management is multidimensional, encompassing disease management and adherence, lifestyle adjustment, cognitive management, and resource utilization (25, 26). While the IBD-Control questionnaire is widely recommended for assessing health-related quality of life (HRQoL) (5) in patients with IBD (including UC), it primarily captures patients' subjective perceptions of symptom burden and disease impact (the outcome) rather than the concrete behavioral processes (the action) that generate these outcomes. To address this conceptual gap, the present study employs the Inflammatory Bowel Disease Self-Management Scale (IBD-SMS) developed by Shang et al. (27). This instrument was specifically constructed to assess IBD patients' SMB across multiple domains, including medication use, symptom monitoring, lifestyle regulation, information seeking, and utilization of medical and social resources, and its reliability and validity have been empirically supported in Chinese IBD populations (which included patients with UC). Building on this foundation, evidence by Zhu et al. (10) has demonstrated SMB measured by the IBD-SMS mediate the relationship between disease activity and IBD control, thereby confirming both the scale's psychometric soundness and its theoretical relevance within the behavioral pathway from disease status to patient-reported disease control.

Therefore, this study adopts a theory-guided approach to:

1) Identify latent profiles of SMB among Chinese patients with UC using the multidimensional IBD-SMS.
2) Examine the association between key theoretical antecedents (e.g., depression, psychological resilience, family communication, socioeconomic status) and membership in these distinct SMB profiles.

## 2 Methods

### 2.1 Study design and settings

This study was conducted at the Department of Gastroenterology at a large tertiary hospital in Zhengzhou, China. The hospital serves a diverse patient population from both urban and rural areas across central China. Participants were recruited from January 2025 to August 2025, primarily from inpatient wards managing patients with UC.

### 2.2 Participants and procedures

Patients who met the following inclusion criteria were eligible for the study: (1) aged ≥18 years; (2) confirmed diagnosis of ulcerative colitis (UC) based on endoscopic and histopathological criteria; (3) ability to complete the questionnaires independently or with assistance; and (4) provision of written informed consent. Exclusion criteria were: (1) diagnosis of Crohn's disease, indeterminate colitis, or other IBD subtypes; (2) inability to complete questionnaires due to cognitive impairment or language barriers; and (3) refusal to participate. The study was approved by the Institutional Ethics Committee of Henan Provincial People's Hospital. The sample size was determined based on recommendations for LPA and requirements for multivariable regression. Simulation studies and prior literature suggest that a minimum sample size of approximately 300 is required to ensure stable class enumeration and reliable parameter estimation (28), and a minimum profile in a large sample needs to include enough individuals (30–60) to support generalizations (29). Allowing for an anticipated 20% rate of incomplete questionnaires, the target sample size was set at approximately 375 participants. A total of 430 adult patients with UC were invited to participate in the study. Of these, 15 patients declined participation or withdrew before completing any questionnaire items; thus, 415 participants were included in the final analyses, exceeding the planned requirement and ensuring adequate statistical power and stability for both LPA and regression models. The patient selection process is illustrated in Supplementary Figure 1. Detailed information on data sources, extraction criteria, and verification procedures is provided in Supplementary Material 2.

### 2.3 Measurement

Patients completed sociodemographic and UC-related variables, such as sex, age, residence, educational level, monthly household income, health insurance reimbursement rate, family structure, primary caregiver, type of health insurance disease duration, and number of hospitalizations. In addition, we investigated the following four variables.

#### 2.3.1 Self-management behavior

SMB were assessed using the IBD Self-management Scale developed by Shang et al. (27). The scale consists of 7 dimensions: medication management, diet management, disease monitoring, emotion management, exercise management, life management, and resource utilization. It includes 36 items scored on a 5-point Likert scale, ranging from 1 (“never”) to 5 (“always”), with total scores ranging from 36 to 180. Higher scores indicate better SMB. This scale was developed for the IBD population, which includes UC, and its reliability and validity have been supported in studies involving Chinese IBD/UC patients. The Cronbach's α of the scale was 0.95, with dimension coefficients ranging from 0.72 to 0.87. The split-half reliability was 0.81, retest reliability was 0.93, Critically, the scale has established criterion-related validity using the Inflammatory Bowel Disease Self-Efficacy Scale (IBD-SES) as the gold standard during its development (*r* = 0.689, *P* < 0.01) (27, 30). In the present study the overall Cronbach's α for the scale was 0.91. Subscale Cronbach's α values were 0.78 0.92. The full item list of the IBD-SMS (in both Chinese and English) is available as Supplementary File 1. To facilitate interpretability of the profile plots, domain scores were transformed to percent of maximum possible (POMP) scores. For each of the seven self-management domains, the POMP score was calculated as: (domain mean score / maximum possible score for that domain) × 100%.

#### 2.3.2 Psychological resilience

The Connor-Davidson Resilience Scale-10 (CD-RISC-10) is a 10-item scale adapted from the original 25-item Connor-Davidson Resilience Scale (31), designed to assess individual psychological resilience levels. The Chinese version demonstrated good psychometric properties, including unidimensionality, internal consistency (Cronbach's α = 0.851), and test-retest reliability (*r* = 0.855). The CD-RISC-10 is widely used in UC research, Sehgal et al. (22) validated its clinical relevance, finding that higher scores significantly correlate with reduced disease activity and surgery risk. In this study Cronbach's α = 0.877.

#### 2.3.3 Depression

The Patient Health Questionnaire-9 (PHQ-9) is a 9-item self-report scale assessing the frequency of depressive symptoms over the past 2 weeks (32). Each item is rated on a 4-point scale from 0 (not at all) to 3 (nearly every day), yielding a total score ranging from 0 to 27. A score of ≥5 indicates potential depressive symptoms, with severity categorized as mild (5–9), moderate (10–14), and severe (15–27). The Chinese version has demonstrated robust reliability and validity in various populations, including general and clinical samples. In this study the PHQ-9 Cronbach's α = 0.876.

#### 2.3.4 Family communication

The Family Communication Scale is a subscale derived from the Family Assessment Device (FAD) and is intended for use with family members aged 12 years and older (33). The FAD framework has been validated in the UC population; specifically, Xu et al. (23) demonstrated that family functioning assessed by the FAD is a key determinant of psychosocial adaptation among Chinese patients with UC. The subscale comprises 9 items. Each item is answered on a 4-point Likert scale (1 = strongly agree to 4 = strongly disagree). Lower scores indicate better family communication. In the present study the subscale demonstrated acceptable internal consistency (Cronbach's α = 0.73).

#### 2.3.5 Social support

The Social Support Rating Scale (SSRS) developed by Xiao (34) was used to assess social support. The SSRS consists of 10 items across three dimensions: objective support, subjective support, and support utilization. Items are scored according to standardized rules, yielding a total score ranging from 12 to 64, with higher scores indicating greater social support. Total scores of ≤ 24 indicate low social support, 24–44 indicate moderate social support, and ≥45 indicate high social support. The scale has demonstrated excellent reliability. Although a generic measure, the SSRS is extensively applied in Chinese UC research to assess the impact of social networks on psychosocial adaptation and demoralization (24). In the present study, the Cronbach's α was 0.89.

### 2.4 Statistical methods

Statistical analyses were performed using IBM SPSS Statistics 26.0 and Mplus 8.3. Continuous variables are presented as mean ± standard deviation (SD) or median (interquartile range) according to distribution; categorical variables are presented as *n* (%). Group differences were tested by one-way ANOVA with Bonferroni *post-hoc* tests or Kruskal–Wallis tests with Dunn's *post-hoc* comparisons for non-normal data, and χ^2^ or Fisher's exact tests for categorical variables. Latent profile analysis (LPA) was conducted using Mplus 8.3 to identify distinct subgroups of SMB. To determine the optimal latent profile solution, we compared models with different parameter specifications. Specifically, we selected a model in which the variances of the seven indicators were freely estimated across classes, and the covariances were fixed to zero under the assumption of local independence (35). Model fit was evaluated using the Akaike Information Criterion (AIC), Bayesian Information Criterion (BIC), sample-size adjusted BIC (aBIC), entropy, the Lo-Mendell-Rubin adjusted likelihood ratio test (LMR-LRT), and the bootstrap likelihood ratio test (BLRT). The final model was selected based on a balance of statistical fit, parsimony, interpretability, and class size. Missing data in LPA were handled using full information maximum likelihood (FIML). Cases excluded prior to analysis (*n* = 15) were those who did not provide any questionnaire data and therefore could not be included in the FIML procedure. To examine factors associated with latent profile membership while accounting for classification uncertainty, we employed the three-step R3STEP procedure implemented in Mplus 8.3 to estimate the effects of covariates (including psychological resilience, depressive symptoms, family communication, social support, and sociodemographic and clinical variables) on class membership. This approach accounts for classification uncertainty by fixing the measurement parameters from the final LPA model and then regressing the latent class variable on the auxiliary variables, yielding unbiased estimates and correct standard errors. The largest class (Low-Monitoring) was set as the reference category. Results are reported as odds ratios (ORs) with 95% confidence intervals (CIs). All tests were two-sided and *p* < 0.05 was considered statistically significant.

### 2.5 Ethics

This cross-sectional study was approved by the Institutional Ethics Committee of Henan Provincial People's Hospital (No. 2025-113) and employed a convenience sampling method. All procedures involving human participants were performed in accordance with relevant guidelines and regulations, including the Declaration of Helsinki. Prior to the study, nurses received comprehensive training on research methods and data collection tools. After screening for eligibility based on inclusion criteria, nurses provided potential participants with written information about the study and obtained their informed consent. Data collection was conducted using structured questionnaires.

## 3 Results

### 3.1 Sample characteristics

A total of 415 patients with UC were included in the analysis. The sample had a mean age of 45.83 years (SD = 13.35) and comprised 259 (62.4%) males. Nearly half of the participants (48.6%) lived in urban areas. The largest education category was college or above (38.1%), and the most frequent monthly household income bracket was CNY 3,000–5,000 (39.8%). The mean psychological resilience score was 28.35 (SD = 6.08), the mean family-communication score was 21.00 (SD = 3.51), and the mean PHQ-9 score was 3.84 (SD = 2.89). Detailed sociodemographic and clinical characteristics are presented in Table 1.

**Table 1:** Sociodemographic and Clinical Characteristics of the Study Cohort (*N* = 415).

Characteristic	Mean ±SD or *n* (%) (*n* = 415)	Characteristic	Mean ±SD or *n* (%) (*n* = 415)
**Age, years**	45.83 ± 13.353	Number of hospitalizations	
Sex		1	134 (41.7%)
Male	259 (62.4%)	2–3	89 (27.7%)
Female	156 (37.6%)	≥4	98 (30.5%)
Residence		Family structure	
Rural	126 (30.4%)	Nuclear family	240 (57.8%)
Town	87 (21%)	15.6-2.2,-1.3249.8ptCouple-only family	175 (42.2%)
Urban	202 (48.6%)	Primary caregiver	
Education level		Spouse	320 (77.1%)
Primary school or below	72 (17.3%)	Other	67 (16.1%)
Junior high or secondary school	118 (28.4%)	15.6-2.2,-1.3249.8ptNone	28 (6.7%)
High school	67 (16.1%)	Ulcerative colitis (UC) disease activity	
College or above	158 (38.1%)	Remission	56 (13.5%)
Monthly household income, CNY		Mild Activity	79 (19%)
<3,000	52 (12.5%)	Moderate Activity	209 (50.4%)
3,000–5,000	165 (39.8%)	15.6-2.2,-1.3249.8ptSevere Activity	71 (17.1%)
5,000–10,000	156 (37.6%)	Type of medical insurance	
>10,000	42 (10.1%)	Resident-based	210 (50.6%)
Medical insurance reimbursement rate		Employee-based	125 (30.1%)
Self-paid	13 (3.1%)	Provincial/Municipal	67 (16.1%)
50– <75%	337 (81.2%)	None	13 (3.1%)
≥75%	65 (15.7%)	**Social support**	28.91 ± 8.134
Disease duration, years		**Psychological resilience**	28.35 ± 6.077
<1	90 (21.8%)	**Family communication**	21.00 ± 3.505
1–5	154 (37.4%)	**Depression**	3.84 ± 2.890
5–10	91 (22.1%)		
>10	77 (18.7%)		

### 3.2 Latent profile analysis of self-management behavior

**Figure 1:**
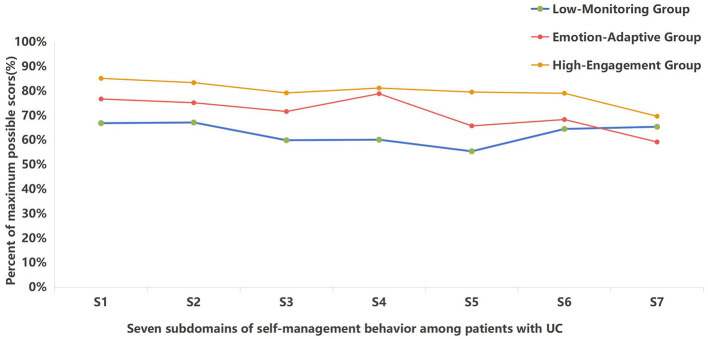
Self-management behavior profiles among patients with UC. S1, medication management; S2, diet management; S3, disease monitoring; S4, emotion regulation; S5, exercise management; S6, daily life management; S7, resource utilization.

Latent class analysis was conducted to identify subgroups of SMB among patients with UC. Model fit indices are summarized in Table 2. Compared with the one-class model, the two-class solution showed significantly improved fit (LMRT *p* < 0.001; BLRT *p* < 0.001) and adequate classification accuracy (entropy = 0.852). The three-class model demonstrated good fit (AIC = 13,548.788; BIC = 13,669.637; aBIC = 13,574.439), with high entropy (0.858) and well-balanced class proportions. The four-class solution yielded slightly lower information criteria (AIC = 13,413.932; BIC = 13,567.007; aBIC = 13,446.423) and a significant BLRT (*p* < 0.001), indicating improved statistical fit. However, examination of the four-class solution revealed that the additional class (comprising 18.554% of the sample) did not represent a substantively distinct behavioral pattern; its profile closely resembled that of the Emotion-Adaptive class but with marginally lower scores on diet management domain, and lacked clear clinical interpretability. Moreover, the improvement in fit indices from three to four classes was modest (ΔBIC = 102.63), and the four-class solution had lower entropy (0.809), suggesting greater classification uncertainty. Given the principle of parsimony, the risk of over-extraction, and the importance of clinical interpretability, the three-class model was retained as the optimal solution. This decision aligns with recommendations to integrate statistical fit, class interpretability, and theoretical meaningfulness in mixture model selection. Percent of maximum possible score of the three latent profiles across the seven SMB subdomains are illustrated in Figure 1.

**Table 2:** Fit indices of latent class models for self-management behavior among patients with UC.

Model	AIC	BIC	aBIC	Entropy	P(LMRT)	P(BLRT)	Class probabilities (%)
1	14,304.262	14,360.658	14,316.233				
2	13,720.116	13,808.739	13,738.927	0.852	<0.001	<0.001	0.60723/0.39277
3	13,548.788	13,669.637	13,574.439	0.858	0.0130	<0.001	0.5397/0.3711/0.0892
4	13,413.932	13,567.007	13,446.423	0.809	0.0189	<0.001	0.1855/0.3181/0.3711/0.1253
5	13,380.123	13,550.456	13,410.789	0.78	0.124	<0.001	0.4517/0.2731/0.1206/0.1052/0.0494

AIC, Akaike Information Criterion; BIC, Bayesian Information Criterion; aBIC, sample-size adjusted Bayesian Information Criterion; LMRT, Lo–Mendell–Rubin likelihood ratio test; BLRT, bootstrap likelihood ratio test.

The three latent classes were subsequently characterized as follows: Profile 1 (Low-Monitoring group), representing 53.97% of patients. Members of this profile demonstrated the lowest standardized scores on disease monitoring and exercise management. Medication and diet management scores were moderate but lower than in the reference profile; resource utilization and daily life management were also comparatively reduced. This pattern indicates limited engagement in symptom surveillance and physical-activity strategies coupled with underuse of external supports. Profile 2 (Emotion-Adaptive group), accounting for 37.11% of the sample, this profile was characterized by relatively high emotion regulation and adherence-related behavior (medication and diet), but lower scores on resource utilization and exercise management. The profile suggests effective internal coping and treatment adherence with inadequate utilization of external resources and activity-based self-management. Profile 3 (High-Engagement group), comprising 8.92% of patients. Members of this smallest profile exhibited higher standardized scores across multiple domains, including medication, diet and emotion regulation, and comparatively better engagement with daily life tasks. Although resource utilization was not uniformly maximal, the overall pattern indicates a proactive and integrated approach to self-management.

### 3.3 Univariate analysis of self-management behavior

Univariate analyses showed statistically significant differences across latent classes for several sociodemographic and clinical variables, including residence, monthly household income, disease duration, social support, psychological resilience, family communication and depressive symptoms (see Supplementary File 2 for full results).

### 3.4 Multinomial logistic regression analysis of latent profiles

Multinomial logistic regression using the R3STEP procedure, with the Low-Monitoring profile as the reference category, revealed distinct patterns of associations (Table 3). For categorical variables, the reference categories were: monthly household income (>10,000 CNY), residence (urban), primary caregiver (none), and UC disease activity (severe).

**Table 3:** Multinomial Logistic Regression (R3STEP) of Factors Associated with Latent Profile Membership (*n* = 415).

Characteristics	Emotion-Adaptive group						High-Engagement group					
	B	Standard error	*t*	*P* value	OR	95% CI	B	Standard error	*t*	*P* value	OR	95% CI
Family communication	−0.064	0.044	−1.455	0.142	0.938	0.861~1.022	0.382	0.194	1.969	0.049	1.466	0.985~2.181
Psychological resilience	−0.100	0.06	−1.667	0.095	0.905	0.805~1.017	0.106	0.041	2.585	0.009	1.112	1.002~2.142
Social support	0.053	0.018	−2.944	0.003	1.054	1.018~1.092	0.097	0.036	2.694	0.007	1.102	1.027~1.182
Depression	−0.156	0.053	−2.944	0.003	0.856	0.770~0.950	−0.618	0.193	−3.202	0.001	0.539	0.369~0.787
Monthly household income												
5 000–10 000	−0.800	0.509	−1.572	0.116	0.449	0.166~1.219	−1.762	0.814	−2.165	0.03	0.172	0.035~0.846
Residence												
Rural	−1.068	0.356	−3.000	0.003	0.344	0.171~0.690	−1.81	0.710	−2.549	0.011	0.164	0.041~0.658
Primary caregiver												
Spouse	0.400	0.160	2.500	0.012	1.492	1.091~2.041	−0.237	0.674	−0.352	0.725	0.789	0.21~2.955
Ulcerative colitis disease activity												
Remission	−1.057	0.468	−2.259	0.024	0.347	0.139~0.87	−2.082	0.953	−2.185	0.029	0.125	0.019~0.807

B, unstandardized regression coefficient; OR, odds ratio; CI, confidence interval. The Low-Monitoring profile served as the reference group.

#### 3.4.1 Emotion-Adaptive vs. Low-Monitoring

Compared with patients in the Low-Monitoring group, those in the Emotion-Adaptive profile reported significantly higher social support (OR = 1.054, 95% CI: 1.018 1.092, *p* = 0.003) and lower depressive symptoms (OR = 0.856, 95% CI: 0.770 0.950, *p* = 0.003). They were likely to have a spouse as the primary caregiver (OR = 1.492, 95% CI: 1.091 2.041, *p* = 0.012), less likely to live in rural areas (OR = 0.344, 95% CI: 0.171 0.690, *p* = 0.003), and less likely to be in disease remission (OR = 0.347, 95% CI: 0.139 0.870, *p* = 0.024). Psychological resilience showed a marginal protective effect that did not reach statistical significance (OR = 0.905, 95% CI: 0.805 1.017, *p* = 0.095), while family communication (*p* = 0.142) and middle income (5,000–10,000 CNY; *p* = 0.116) were not significantly associated with membership in this profile.

#### 3.4.2 High-Engagement vs. Low-Monitoring

Patients in the High-Engagement profile, compared with the Low-Monitoring group, demonstrated significantly higher psychological resilience (OR = 1.112, 95% CI: 1.002 2.142, *p* = 0.009), higher social support (OR = 1.102, 95% CI: 1.027 1.182, *p* = 0.007). Better family communication was associated with membership in the High-Engagement profile, although this association was of borderline statistical significance (OR = 1.466, 95% CI: 0.985 2.181, *p* = 0.049). Conversely, they had lower depressive symptoms (OR = 0.539, 95% CI: 0.369 0.787, *p* = 0.001). Several factors were associated with reduced odds of being in the High-Engagement group: rural residence (OR = 0.164, 95% CI: 0.041 0.658, *p* = 0.011), monthly household income of 5,000–10,000 CNY (OR = 0.172, 95% CI: 0.035 0.846, *p* = 0.030), and being in disease remission (OR = 0.125, 95% CI: 0.019 0.807, *p* = 0.029). Having a spouse as the primary caregiver was not significantly different between the two groups (*p* = 0.725) (see Table 3).

## 4 Discussion

This study identified significant heterogeneity in SMB among Chinese patients with UC. LPA delineated three clinically distinct behavioral profiles: Low-Monitoring (53.97%), Emotion-Adaptive (37.11%), and High-Engagement (8.92%). Our identification of discrete SMB profiles is consistent with recent latent profile work across chronic conditions showing that self-management is not a unitary construct but clusters into qualitatively different patterns of strengths and deficits (36, 37). However, this study extends the existing literature by contextualizing these behavioral phenotypes within the specific clinical demands of UC. Crucially, these behavioral phenotypes offer a novel lens through which to understand divergent patient pathways toward the contemporary treat-to-target goal of Comprehensive Disease Control (CDC). As defined by international Delphi consensus (38), CDC in UC requires the simultaneous achievement of clinical remission, endoscopic remission, histologic remission, control of patient-important symptoms (e.g., bowel urgency and fatigue), and restoration of health-related quality of life. The High-Engagement Profile represents the optimal behavioral archetype for achieving and sustaining CDC. Its proactive, multi-domain management strategy (from medication to resource utilization) provides the foundational behavioral substrate necessary for maintaining deep remission and high functional status across all CDC dimensions. This group parallels the high self-management profiles reported internationally, and they likely exhibit better psychosocial adaptation and quality of life (20), which indicates the successful integration of disease management into daily life, forming a stable and healthy behavioral system. The Low-Monitoring Profile identifies a high-risk behavioral phenotype vulnerable to CDC failure. Profound deficits in disease monitoring (S3, 69.91%) directly jeopardize the core CDC pillars of endoscopic and histologic remission, mirrors “low self-management” clusters found in other chronic disease studies (18). This pattern predisposes patients to delayed recognition of subclinical inflammation and clinical relapse, positioning this group as the highest priority for structured, nurse-led intervention. The novel Emotion-Adaptive Profile illuminates a potential pathway to discrepant CDC outcomes, a similar pattern has been noted whereby psychosocial coping strategies (vs. strict regimen adherence) influence outcomes (39). While strong internal emotion regulation is a key asset for preserving health-related quality of life and psychosocial function, the relative neglect of proactive health behavior and external resource utilization may undermine long-term maintenance of objective inflammatory control. This profile highlights the need for interventions that bridge internal coping skills with outward disease-management actions.

Following the Middle-Range Explanatory Theory of SMB (21), we included all theoretically specified antecedents simultaneously in the multivariable model, avoiding the limitations of data-driven variable selection (40). This approach ensures that the observed associations reflect the joint contribution of psychological, familial, and sociodemographic factors as posited by the theoretical framework. The regression results reveal the differential impact of various factors on class membership, constructing a multi-layered influence framework.

### 4.1 Psychological factors

In the present study, psychological resilience showed differential associations with profile membership. Compared with the Low-Monitoring group, patients in the High-Engagement profile demonstrated significantly higher resilience (OR = 1.112, *p* = 0.009), whereas the difference between the Emotion-Adaptive and Low-Monitoring groups did not reach statistical significance (OR = 0.905, *p* = 0.095), with a trend toward lower resilience in the Emotion-Adaptive group. This pattern suggests that higher psychological resilience was associated with patients who adopt a comprehensive, multi-domain approach to self-management, rather than those who rely primarily on emotion regulation strategies. These findings align with a seminal study demonstrating that high psychological resilience in UC patients is independently associated with lower disease activity, better quality of life, and fewer surgeries, underscoring the robust association between psychological resilience and favorable disease outcomes (22, 41). Thus, resilience-enhancing interventions may warrant investigation in self-management support, as resilience is associated with the psychological resources that may support patients in enduring flare-ups, emotional disturbances, and the complex demands of long-term treatment behavior (42).

Consistent with Van Den Houte et al. (17), the present study found that elevated depressive symptoms were associated with lower odds of membership in both the Emotion-Adaptive (OR = 0.856, *p* = 0.003) and High-Engagement (OR = 0.539, *p* = 0.001) profiles compared with the Low-Monitoring group. Prospective clinical evidence corroborates this association: depressed in-patients demonstrate lower self-management competence, particularly in domains that require planning, sustained motivation and task execution (43). Cognitive behavioral models of symptom perpetuation (44) further suggest that, although biological inflammation initiates symptoms, the persistence and perceived severity of symptoms are shaped by interacting cognitive, emotional and behavioral processes; depression is associated with maladaptive cognitive biases and is associated with reduced cognitive and motivational resources. Collectively, these complementary perspectives suggest that depressive symptoms are strongly linked to poorer SMB, in a manner that goes beyond simple co-occurrence. Accordingly, interventions are likely to be most effective when they combine mood-directed treatment and psychological resilience building with behavioral strategies that lighten cognitive load in clinic encounters (for example, simplified shared decision-making tools), restructure patients' activation and self-efficacy to increase motivation, persistence, and the translation of intentions into sustained SMB, and concurrently build self-management competencies through skills training and scaffolded supports (45, 46).

### 4.2 Family system factors

Family communication was associated with membership in the High-Engagement profile, though this association reached only borderline statistical significance (OR = 1.466, 95% CI: 0.985 2.181, *p* = 0.049). It was not associated with the Emotion-Adaptive profile (*p* = 0.142) when compared with the Low-Monitoring group. Given the confidence interval crossing the null, this finding should be interpreted with caution. This finding suggests that open and effective family communication may be particularly important for supporting comprehensive, multi-domain self-management, rather than emotion-focused coping alone. This may be because emotion-focused coping relies more on individual internal resources, whereas comprehensive self-management requires external support and coordination that effective family communication can provide. In well-functioning families, open dialogue, emotional validation, practical reminders, and collaborative problem-solving was associated with lower psychological distress and are associated with better SMB. Studies indicate that good family functioning can improve anxiety and depression by boosting patient self-esteem (47), and practical support from family is directly linked to improved treatment adherence and quality of life (48). These connections, established in the broader IBD population, warrant further exploration specifically in UC, an important subtype.

Compared with the Low-Monitoring group, patients in both the Emotion-Adaptive (OR = 1.054, *p* = 0.003) and High-Engagement (OR = 1.102, *p* = 0.007) profiles reported significantly higher social support. This finding indicates that social support is independently associated with more adaptive self-management patterns, even after accounting for psychological resilience, depressive symptoms, and family communication. The direct association observed in this study aligns with a substantial body of evidence demonstrating the importance of social support in chronic disease management (49, 50). Social support was associated with self-management through multiple pathways (51). In the context of UC, where disease course is unpredictable and treatment regimens can be complex, a supportive social network was associated with patients navigating challenges and maintain engagement with self-management activities over time. Thus, qualitative work exploring how patients perceive and make use of different forms of support could identify actionable targets for nursing interventions.

### 4.3 Socioeconomic context factors

Rural residence was associated with lower odds of membership in both more engaged profiles (Emotion-Adaptive: OR = 0.344, *p* = 0.003; High-Engagement: OR = 0.164, *p* = 0.011), while middle-level household income (5,000–10,000 RMB) was specifically associated with lower odds of belonging to the High-Engagement profile (OR = 0.172, *p* = 0.030) but not the Emotion-Adaptive profile (*p* = 0.116). These findings suggest that geographic and economic factors were associated with adopting comprehensive SMB. Rural patients may face systemic barriers such as fewer healthcare providers, geographic isolation, and lower insurance reimbursement rates, which may be related to access to disease monitoring and care continuity (52, 53). Interestingly, having a spouse as the primary caregiver was associated with higher odds of being in the Emotion-Adaptive profile (OR = 1.492, *p* = 0.012) but not the High-Engagement profile (*p* = 0.725), suggesting that spousal support may be related to emotion-focused coping but may not be sufficient to promote comprehensive self-management engagement. Meanwhile, patients with UC from middle-income households appear particularly vulnerable to suboptimal SMB, as evidenced by their lower likelihood of belonging to the High-Engagement profile. Although their nominal income may place them above the lowest socioeconomic strata, many middle-income households experience substantial recurring financial obligations (for example, mortgage payments, tuition, or other large fixed costs) that create pronounced financial strain. Evidence from Osborn (54) indicates that perceived financial strain, which measured as difficulty paying bills, is more strongly associated with medication nonadherence and poorer self-rated health than absolute income. In this socioeconomic context, expenditures and time devoted to long-term health maintenance are frequently subordinated to urgent family needs (housing, childcare, education), so that continued adherence to preventive medications, dietary regimens and clinic follow-up becomes a lower priority or an unaffordable “luxury” (55, 56). Empirical studies of “financial toxicity” in Chinese patients document exactly such effects across chronic conditions and cancer care (57). The PACE telemedicine program (58) demonstrates that a hub-and-spoke, multidisciplinary virtual care model can substantially reduce wait times and out-of-pocket travel costs for rural and underserved UC patients; embedding nurse-led SMB coaching, rapid-access triage and remote symptom monitoring into such models may mitigate the economic and geographic barriers associated with poor self-management.

### 4.4 Patients in clinical remission

A notable finding was that patients in clinical remission were significantly less likely to belong to the Emotion-Adaptive (OR = 0.347, *p* = 0.024) and High-Engagement (OR = 0.125, *p* = 0.029) profiles compared with the Low-Monitoring group. In other words, patients in remission were more likely to be classified in the least engaged self-management profile. This phenomenon may largely related to a symptom-centric understanding of the disease. As Huisman (59) showed, over 55% of patient-directed websites define remission solely by symptom control, and a U.S. survey revealed that most UC patients define remission simply as the reduction or absence of symptoms (60). However, multiple studies (61, 62) demonstrate substantial discordance between patient-reported outcomes and objective disease activity; symptomatic improvement often precedes full mucosal healing, and elevated fecal calprotectin can predict relapse despite clinical quiescence. Consequently, patients may feel well while significant subclinical inflammation persists, leading to critical lapses in surveillance (63).

From a theoretical perspective, this “remission paradox” may be interpreted through symptom perception. Within the Health Belief Model framework (64), acute symptoms act as continuous somatic cues. The loss of these symptoms during remission significantly reduces patients' perceived susceptibility and severity of the disease, thereby weakening their “cues to action.” Consequently, this lowers their motivation to continue behaviors whose benefits are invisible and delayed, such as maintenance medication, biomarker monitoring, and regular exercise (11). To address this paradox, self-management support strategies should be also implemented at clinical remission period. Practical interventions should include structured “remission-transition” counseling led by UC nurses. By utilizing visual biomarker trends (e.g., graphing fecal calprotectin levels), nurses can make subclinical inflammation “visible,” and effectively recalibrating the patient's symptom perception (65, 66). Integrating theory-driven digital self-monitoring applications that provide gamified feedback with proactive, nurse-led telehealth check-ins may enhance patients' motivation and adherence to SMB in UC care (67).

## 5 Limitation and clinical implications

This study has several important limitations. First, the cross-sectional design precludes causal inferences regarding the relationships between self-management profiles and associated psychosocial or clinical factors. Second, potential selection bias may limit the external validity of our results. The study population was restricted to patients with UC from a single regional tertiary center in East China. Notably, as participants were recruited primarily from inpatient wards, the cohort skewed significantly toward higher disease severity compared to real-world datasets like the UK IBD Registry (68). This facility-related bias may partially explain the relatively small size of the High-Engagement profile (8.92%) and the reduced precision of some parameter estimates. Therefore, the identified latent profiles should be interpreted as reflecting SMB in a selectively severe population and may not represent the broader UC community, including outpatients, patients in sustained remission, or individuals with different sociocultural backgrounds and disease types (e.g., Crohn's disease). Third, reliance on self-report measures introduces potential recall and social-desirability biases. Furthermore, while the IBD-SMS exhibits strong psychometric properties in mixed Chinese IBD cohorts, its measurement invariance across UC and CD subtypes remains to be formally established. The absence of concurrent standardized international patient-reported outcomes (PROs), such as the IBD-Control questionnaire (23), also restricts direct cross-cultural comparisons. Finally, because the latent profiles were derived solely from self-management domains, excluding objective clinical symptoms, biomarkers, or broader quality-of-life measures, they represent preliminary behavioral phenotypes. Future research should leverage joint trajectory models in larger, more diverse cohorts to integrate these multifaceted clinical indicators, thereby confirming comprehensive clinical-behavioral typologies.

Despite these constraints, the identified typology has clear clinical implications for nursing. These findings suggest the utility of integrating psychological screening (e.g., resilience and distress) alongside behavioral tracking to identify patients at higher risk of poor disease control. Recognizing these patterns may assist healthcare providers in prioritizing resources for patients who demonstrate a cluster of suboptimal behaviors. Furthermore, the strong association observed between higher resilience and profiles suggests that psychological status is a relevant marker of a patient's capacity to navigate the complexities of UC treatment. These descriptive insights provide a basis for the future development of personalized management strategies tailored to the specific behavioral phenotypes prevalent in the UC population. Future multicenter, longitudinal and intervention studies that include objective adherence and cost-effectiveness outcomes are needed to validate the typology and to determine whether profile-guided nursing care improves clinical and health-service outcomes.

## 6 Conclusion

This study clarifies the heterogeneity of SMB among Chinese patients with UC through LPA, identifying a behavioral typology that serves as a critical mechanism for optimizing patient-reported outcomes. The findings highlight that self-management in UC is not a uniform construct but rather a multidimensional pattern that varies across different patient subgroups.

## Data Availability

The original contributions presented in the study are included in the article/Supplementary material, further inquiries can be directed to the corresponding author.
