# Supplementary material for: Latent profiles of self-management behavior and associated factors among Chinese patients with ulcerative colitis
Source: Front Public Health. 2026 Apr 17;14:1749767. doi: 10.3389/fpubh.2026.1749767 (PMC13132808; doi:10.3389/fpubh.2026.1749767)
Supplement: Supplementary file 1 [file Supplementary_file_1.docx]

**炎症性肠病病人自我管理行为量表**

本问卷是用来调查您疾病自我管理行为。请您根据自身实际情况在适当的方格里打“√”。

| **条目** | **从来不** | **很少** | **有时** | **经常** | **总是** |
| --- | --- | --- | --- | --- | --- |
| 1.您了解自己所服用的炎症性肠病相关药物的用法、用量、作用、副作用及注意事项吗? | 1 | 2 | 3 | 4 | 5 |
| 2.您能遵从医护人员的建议正确使用治疗炎症性肠病的药物吗?(按时、按剂量、按疗程) |  |  |  |  |  |
| 3.当炎症性肠病疾病症状控制良好，您仍能按要求服药吗? |  |  |  |  |  |
| 4.您能够及时发现治疗炎症性肠病相关药物的不良反应吗? |  |  |  |  |  |
| 5.当您发现所用药物有不良反应或病情变化时，会及时和医生沟通，遵医嘱调整用药吗? |  |  |  |  |  |
| 6.您能控制甜食(巧克力、口香糖、可乐等)、肉类、高脂食物的摄入吗? |  |  |  |  |  |
| 7.您能控制含食品添加剂、防腐剂的休闲食品的摄入吗? |  |  |  |  |  |
| 8.您能控制快餐或外卖等食物的摄入吗? |  |  |  |  |  |
| 9.您会注重食物的烹饪方式吗?(如以煮、蒸为主，少油煎、炸) |  |  |  |  |  |
| 10.您会定期监测自身体重进行营养管理吗? |  |  |  |  |  |
| 11.您能控制生、冷、硬、辛辣刺激(如辣椒、浓茶、咖啡)、油腻、粗纤维食物(如玉米、笋、韭菜、芹菜、花菜)、海鲜等的摄入吗? |  |  |  |  |  |
| 12.您会注意饮食摄入的营养均衡吗? |  |  |  |  |  |
| 13.您能通过记录饮食日记的方式排除自己不耐受的食物吗? |  |  |  |  |  |
| 14.您会避免暴饮暴食吗? |  |  |  |  |  |
| 15.您会密切观察自己大便的次数及性状吗? |  |  |  |  |  |
| 16.您会遵医嘱定期来医院复诊吗? |  |  |  |  |  |
| 17.您会密切观察自己的腹部症状吗?(如腹痛、腹部肿块) |  |  |  |  |  |
| 18.您能及时识别疾病复发表现吗? |  |  |  |  |  |
| 19.情绪低落时，您能鼓励自己尽快振作起来吗? |  |  |  |  |  |
| 20.情绪低落时，您会寻求一些缓解情绪的方法吗(如散步、听音乐、看电影等)? |  |  |  |  |  |
| 21.您会努力调动自身积极情绪来促进疾病缓解吗? |  |  |  |  |  |
| 22.当您有“自己无用”的想法，会尝试尽力改变吗? |  |  |  |  |  |
| 23.当您有“自己是家人负担”的想法时，会尝试尽力改变吗? |  |  |  |  |  |
| 24.当出现疾病相关并发症时，您能保持情绪稳定吗? |  |  |  |  |  |
| 25.您能根据自身情况或医护人员建议选择运动项目、运动强度及时间吗? |  |  |  |  |  |
| 26.您能根据病情变化调整运动形式或强度吗? |  |  |  |  |  |
| 27.您锻炼后会合理补充水分和盐分吗? |  |  |  |  |  |
| 28.您能保持规律的生活，每天早睡早起，保证充足的睡眠吗? |  |  |  |  |  |
| 29.您能避免过度疲劳吗? |  |  |  |  |  |
| 30.您能平衡工作、活动和休息吗? |  |  |  |  |  |
| 31.您会参加一些社交活动，维持良好的社会关系吗? |  |  |  |  |  |
| 32.情绪低落时，您会向家人或朋友倾诉，寻求安慰或帮助吗? |  |  |  |  |  |
| 33.您会与病友交流疾病相关信息吗？ |  |  |  |  |  |
| 34.您能通过网络、书籍等途径主动学习炎症性肠病  相关的疾病保健知识吗？ |  |  |  |  |  |
| 35.您能识别所获取信息的可靠性吗？ |  |  |  |  |  |
| 36.当疾病复发或出现并发症时，您会寻求家庭成员  的支持和帮助吗？ |  |  |  |  |  |

### ****Self-Management Behavior Scale for Inflammatory Bowel Disease (IBD) Patients****

This questionnaire investigates your self-management behaviors related to IBD. Please mark "√" in the box that best represents your actual situation.

| **Item** | **Never**  **(1)** | **Rarely**  **(2)** | **Sometimes**  **(3)** | **Often**  **(4)** | **Always**  **(5)** |
| --- | --- | --- | --- | --- | --- |
| 1. Are you familiar with the usage, dosage, effects, side effects, and precautions of your IBD medications? |  |  |  |  |  |
| 2. Can you follow healthcare providers' instructions in using IBD medications (on time, correct dose, complete course)? |  |  |  |  |  |
| 3. Can you continue taking your medication as required when symptoms are well controlled? |  |  |  |  |  |
| 4. Are you able to detect adverse reactions to IBD medications in time? |  |  |  |  |  |
| 5. Do you communicate promptly with your doctor when adverse effects or disease progression occurs, and adjust medication accordingly? |  |  |  |  |  |
| 6. Can you control your intake of sweets (e.g., chocolate, gum, cola), meat, and high-fat foods? |  |  |  |  |  |
| 7. Can you limit intake of processed snacks with additives or preservatives? |  |  |  |  |  |
| 8. Can you restrict fast food or takeaway consumption? |  |  |  |  |  |
| 9. Do you pay attention to healthy cooking methods (e.g., boiling/steaming instead of frying)? |  |  |  |  |  |
| 10. Do you regularly monitor your weight to manage nutrition? |  |  |  |  |  |
| 11. Can you control consumption of raw, cold, hard, spicy, greasy, fibrous foods (e.g., corn, bamboo shoots, leeks, celery, cauliflower), seafood, etc.? |  |  |  |  |  |
| 12. Do you ensure a nutritionally balanced diet? |  |  |  |  |  |
| 13. Do you keep a food diary to identify intolerances? |  |  |  |  |  |
| 14. Do you avoid binge eating? |  |  |  |  |  |
| 15. Do you closely monitor your bowel movements (frequency and appearance)? |  |  |  |  |  |
| 16. Do you attend regular follow-up visits as advised by your doctor? |  |  |  |  |  |
| 17. Do you monitor your abdominal symptoms (e.g., pain, lumps)? |  |  |  |  |  |
| 18. Are you able to recognize signs of disease relapse promptly? |  |  |  |  |  |
| 19. When feeling low, can you encourage yourself to recover emotionally? |  |  |  |  |  |
| 20. When feeling low, do you seek emotional relief (e.g., walking, music, movies)? |  |  |  |  |  |
| 21. Do you make efforts to cultivate positive emotions to aid recovery? |  |  |  |  |  |
| 22. When you feel "useless", do you try to change this mindset? |  |  |  |  |  |
| 23. When you feel like a burden to your family, do you try to overcome that thought? |  |  |  |  |  |
| 24. When complications occur, can you remain emotionally stable? |  |  |  |  |  |
| 25. Can you choose appropriate physical activity (type, intensity, duration) based on your condition or professional advice? |  |  |  |  |  |
| 26. Can you adjust your exercise routines according to disease changes? |  |  |  |  |  |
| 27. Do you rehydrate and replenish electrolytes appropriately after exercise? |  |  |  |  |  |
| 28. Do you maintain a regular schedule (early to bed and rise, sufficient sleep)? |  |  |  |  |  |
| 29. Can you avoid overexertion? |  |  |  |  |  |
| 30. Can you balance work, activities, and rest? |  |  |  |  |  |
| 31. Do you participate in social activities and maintain healthy relationships? |  |  |  |  |  |
| 32. When feeling down, do you confide in family or friends for comfort or help? |  |  |  |  |  |
| 1. Do you exchange disease-related information with fellow patients? |  |  |  |  |  |
| 1. Can you proactively learn about health maintenance knowledge related to Inflammatory Bowel Disease through channels like the internet or books on your own initiative? |  |  |  |  |  |
| 1. Are you able to judge the reliability of the information you obtain? |  |  |  |  |  |
| 1. When your disease recurs or complications arise, do you seek support and help from family members? |  |  |  |  |  |
