# Supplementary material for: Latent profiles of self-management behavior and associated factors among Chinese patients with ulcerative colitis
Source: Front Public Health. 2026 Apr 17;14:1749767. doi: 10.3389/fpubh.2026.1749767 (PMC13132808; doi:10.3389/fpubh.2026.1749767)
Supplement: Supplementary file 2 [file Supplementary_file_2.docx]

This supplementary material provides detailed information on the data sources, variable definitions, extraction procedures, and disease activity assessment methods used to generate the data presented in Table 1 of the main manuscript. The purpose is to ensure transparency regarding the construction of the analytical cohort and the classification of disease activity, addressing reviewer queries about data integrity and reproducibility.

**1.All data were collected from two primary sources:**

| Data Source | Description | Variables Collected |
| --- | --- | --- |
| **Electronic Medical Records (EMR)** | Hospital's inpatient electronic medical record system | Age, sex, residence, disease duration, number of hospitalizations, UC disease activity (Mayo score components), type of medical insurance, insurance reimbursement rate |
| **Patient-Reported Questionnaires** | Self-administered paper questionnaires completed during hospitalization | Education level, monthly household income, family structure, primary caregiver, psychological resilience (CD-RISC-10), depressive symptoms (PHQ-9), family communication (FAD subscale), social support (SSRS), self-management behaviors (IBD-SMS) |

Data extraction was performed by two trained research nurses between January 2025 and August 2025. All extracted data were double-entered into a secure research database and verified for consistency.

**2.Variable Definitions and Extraction Criteria**

| Variable | Definition/Categories | Source | Coding/Notes |
| --- | --- | --- | --- |
| Age | Age in years at time of enrollment | EMR | Continuous variable |
| Sex | Biological sex | EMR | 1 = Male, 2 = Female |
| Residence | Place of residence based on household registration | Patient questionnaire | 1 = Rural, 2 = Town, 3 = Urban |
| Education Level | Highest level of education completed | Patient questionnaire | 1 = Primary school or below, 2 = Junior high or secondary school, 3 = High school, 4 = College or above |
| Monthly Household Income | Total monthly household income in CNY | Patient questionnaire | 1 = <3,000, 2 = 3,000–5,000, 3 = 5,000–10,000, 4 = >10,000 |
| Family Structure | Household composition | Patient questionnaire | 1 = Nuclear family (parents and unmarried children), 2 = Couple-only family |
| Primary Caregiver | Main person providing care and support | Patient questionnaire | 1 = Spouse, 2 = Other (children, parents, relatives), 3 = None |

#### 3 Clinical Variables

| Variable | Definition/Categories | Source | Coding/Notes |
| --- | --- | --- | --- |
| UC Diagnosis | Confirmed diagnosis of ulcerative colitis based on endoscopic and histopathological criteria | EMR (endoscopy and pathology reports) | Only patients with definitive UC diagnosis included; Crohn's disease and indeterminate colitis excluded |
| Disease Duration | Time since initial UC diagnosis | EMR | 1 = <1 year, 2 = 1–5 years, 3 = 5–10 years, 4 = >10 years |
| Number of Hospitalizations | Total number of UC-related hospitalizations (including current) | EMR | 1 = 1, 2 = 2–3, 3 = ≥4 |
| Type of Medical Insurance | Primary health insurance coverage | EMR | 1 = Resident-based, 2 = Employee-based, 3 = Provincial/Municipal, 4 = None (self-paid) |
| Insurance Reimbursement Rate | Proportion of medical expenses covered by insurance | EMR | 1 = Self-paid, 2 = 50–<75%, 3 = ≥75% |

### 4. Disease Activity Assessment

#### 4.1 Assessment Tool: Mayo Clinic Score

Disease activity was assessed using the Mayo Clinic Score (also known as the Ulcerative Colitis Disease Activity Index), which consists of four components:

| Component | Description | Score Range | Scoring Criteria |
| --- | --- | --- | --- |
| Stool Frequency | Number of stools per day compared to normal | 0–3 | 0 = Normal number of stools 1 = 1–2 stools more than normal 2 = 3–4 stools more than normal 3 = 5 or more stools more than normal |
| Rectal Bleeding | Amount of blood in stools | 0–3 | 0 = No blood seen 1 = Streaks of blood with stool less than half the time 2 = Obvious blood with stool most of the time 3 = Blood alone passed |
| Endoscopic Findings | Mucosal appearance on sigmoidoscopy/colonoscopy | 0–3 | 0 = Normal or inactive disease 1 = Mild (erythema, decreased vascular pattern, mild friability) 2 = Moderate (marked erythema, absent vascular pattern, friability, erosions) 3 = Severe (spontaneous bleeding, ulceration) |
| Physician Global Assessment | Physician's overall assessment based on the above three criteria and other clinical findings | 0–3 | 0 = Normal 1 = Mild disease 2 = Moderate disease 3 = Severe disease |

Total Mayo Score = Sum of all four components (range 0–12)

#### 4.2 Disease Activity Classification

Based on the total Mayo score, patients were classified into four disease activity categories as follows:

| Disease Activity Category | Total Mayo Score | Additional Criteria | n (%) in Current Study |
| --- | --- | --- | --- |
| Remission | ≤2 | No individual subscore >1 | 56 (13.5%) |
| Mild Activity | 3–5 | - | 79 (19.0%) |
| Moderate Activity | 6–10 | - | 209 (50.4%) |
| Severe Activity | 11–12 | - | 71 (17.1%) |

**4.3 Assessment Procedures**

Timing: Disease activity was assessed at the time of enrollment (within 48–96 hours of admission).

Assessors: Endoscopic subscores were determined by attending gastroenterologists based on the most recent colonoscopy (performed within 1 week prior to enrollment). Stool frequency, rectal bleeding, and physician global assessment were evaluated by trained research nurses through patient interview and clinical record review.

Quality Control: All assessments were reviewed by a senior gastroenterologist to ensure consistency. Discrepancies were resolved by consensus.

### 5. Disease Subtype Confirmation

To ensure the analytical cohort consisted exclusively of UC patients, the following verification procedures were applied:

| Step | Procedure | Purpose |
| --- | --- | --- |
| 1 | Initial screening of EMR for diagnosis codes | Identify potential UC cases |
| 2 | Review of endoscopic reports | Confirm characteristic UC findings (continuous mucosal inflammation starting from rectum, uniform involvement, absence of skip lesions) |
| 3 | Review of histopathological reports | Confirm histologic features consistent with UC (crypt abscesses, mucosal inflammation, no granulomas) |
| 4 | Final verification by gastroenterologist | Resolve ambiguous cases; exclude Crohn's disease, indeterminate colitis, or other IBD subtypes |
