# Supplementary material for: Latent profiles of self-management behavior and associated factors among Chinese patients with ulcerative colitis
Source: Front Public Health. 2026 Apr 17;14:1749767. doi: 10.3389/fpubh.2026.1749767 (PMC13132808; doi:10.3389/fpubh.2026.1749767)
Supplement: Supplementary file 3 [file Image_1.pdf]

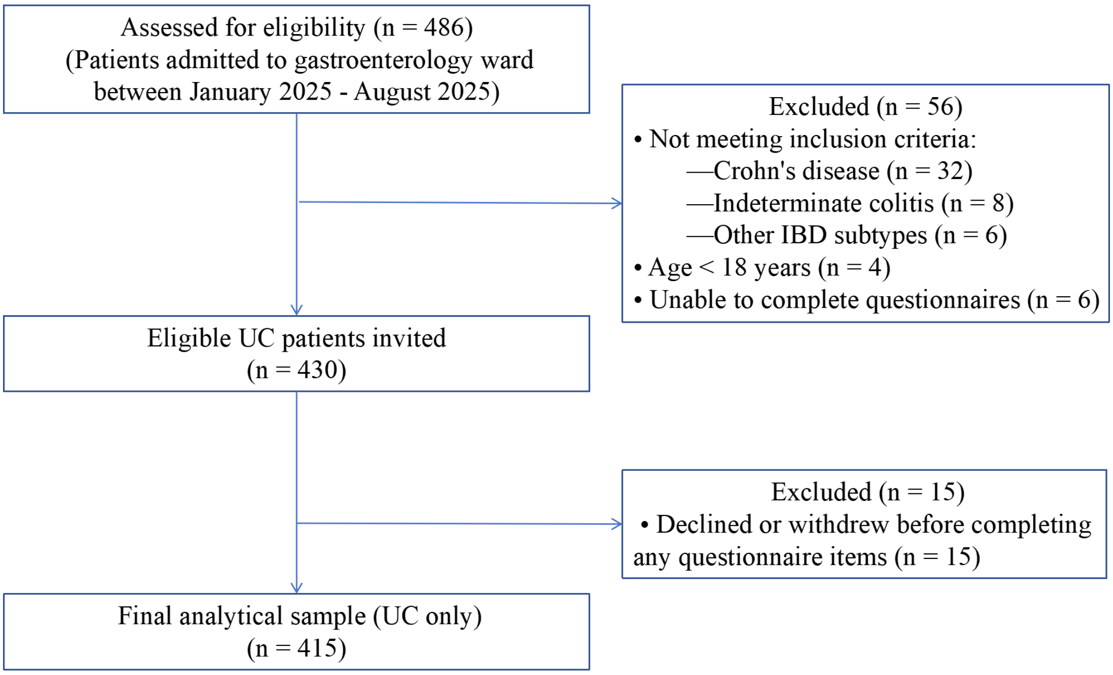

Supplementary Figure 1. Patient flow diagram illustrating the selection process for the final analytical sample.

Of 486 patients initially assessed for eligibility, 56 were excluded due to not meeting inclusion criteria (including 32 with Crohn's disease, 8 with indeterminate colitis, and 6 with other IBD subtypes). A total of 430 eligible UC patients were invited to participate, of whom 15 declined or withdrew before completing any questionnaire items. The final analytical sample comprised 415 patients with confirmed UC, all of whom were included in the latent profile analysis and R3STEP regression.
